# Occurrence of *Fusarium langsethiae* and T-2 and HT-2 Toxins in Italian Malting Barley

**DOI:** 10.3390/toxins8080247

**Published:** 2016-08-20

**Authors:** Caterina Morcia, Giorgio Tumino, Roberta Ghizzoni, Franz W. Badeck, Veronica M.T. Lattanzio, Michelangelo Pascale, Valeria Terzi

**Affiliations:** 1 Genomics Research Centre (CREA-GPG), Council for Agricultural Research and Economics, Via San Protaso 302, 29017 Fiorenzuola d’Arda (PC), Italy; caterina.morcia@crea.gov.it (C.M.); giorgiotumino@hotmail.it (G.T.); roberta.ghizzoni@crea.gov.it (R.G.); franz-werner.badeck@crea.gov.it (F.W.B.); 2 Institute of Sciences of Food Production (ISPA), National Research Council of Italy (CNR), via G. Amendola 122/O, 70126 Bari, Italy; veronica.lattanzio@ispa.cnr.it (V.M.T.L.); michelangelo.pascale@ispa.cnr.it (M.P.)

**Keywords:** T-2 toxin, HT-2 toxin, *Fusarium langsethiae*, toxigenic fungi, Fusarium Head Blight, barley, monitoring

## Abstract

T-2 and HT-2 toxins are two of the most toxic members of type-A trichothecenes, produced by a number of *Fusarium* species. The occurrence of these mycotoxins was studied in barley samples during a survey carried out in the 2011–2014 growing seasons in climatically different regions in Italy. The percentage of samples found positive ranges from 22% to 53%, with values included between 26 and 787 μg/kg. The percentage of samples with a T-2 and HT-2 content above the EU indicative levels for barley of 200 μg/kg ranges from 2% to 19.6% in the 2011–2014 period. The fungal species responsible for the production of these toxins in 100% of positive samples has been identified as *Fusarium langsethiae*, a well-known producer of T-2 and HT-2 toxins. A positive correlation between the amount of *F. langsethiae* DNA and of the sum of T-2 and HT-2 toxins was found. This is the first report on the occurrence of *F. langsethiae*—and of its toxic metabolites T-2 and HT-2—in malting barley grown in Italy.

## 1. Introduction

Barley is a widespread crop that ranks fourth among the world’s cereals for the importance of its contribution to feed and food production. About 80%–90% of barley grain yield is earmarked for livestock feed and about 10% is transformed into malt for many brewing, distilling and baking applications [1]. Malting is a controlled process of germination that begins with the kernel imbibition, followed by the modification period. During modification a battery of enzymes (glucanases, pentosanases, phosphatases, phytases, proteases, amylases) alters the endosperm’s structure and converts starch into sugars and proteins into amino acids. The result is a highly nutritious medium for subsequent fermentations. Malting barley varieties are specialty crops, considered the raw materials par excellence for malt production because they are specifically bred to optimize many qualitative and technological properties at the grain, malt and wort levels. Quality requirements for malting barley are strictly and directly related to processing efficiency and product quality in the malting and brewing industries. The presence of diseased kernels is a strong negative factor: fungal spores can in fact be carried through the malting process and affect both the beer quality and safety. The malt contamination with filamentous fungi, such as *Fusarium* spp., is now recognized as the most important factor involved in primary gushing, i.e., an uncontrolled escape of wet foam when opening a beer bottle. This phenomenon is not caused by high temperature or shaking, but is caused by the presence of fungal hydrophobins [2,3,4]. Moreover, *Fusarium* spp. can produce not only hydrophobins, but even mycotoxins, different in structure and biological function, that are synthesized by different mechanisms at different stages of the fungal life cycle [4]. Mycotoxin contamination of malting barley grains is a major problem from a safety point of view because these fungal secondary metabolites affect human health. Reducing the level of mycotoxin contamination in both malting and feed barley is therefore of high priority. Several different strategies can be employed to mitigate the problem, ranging from breeding resistant varieties to the use of synthetic and natural molecules for crop protection, from the bio-control of fungal populations to the adoption of technological solutions [5]. Even monitoring activities can contribute effectively to the control of the mycotoxins problem through the early identification of fungal attacks, the characterization of the fungal species present and of their infection levels on plants and grains and the identification of emerging fungal species.

Recent surveys carried out in Europe have depicted a complex and varied situation for mycotoxin occurrence and mycotoxigenic fungal colonization in barley. Kirincic et al. [6] evaluated the mycotoxin occurrence in different cereals and cereal-based products that have been sampled in the frame of official control activities in Slovenia during the years 2008–2012. These authors found that barley products are most frequently contaminated with deoxynivalenol (DON) and zearalenone (ZON). In barley seeds harvested in different fields located in six Croatian regions during 2011, DON was found to be the most frequent mycotoxin, followed by T-2 toxin and fumonisins (FUM) [7]. In Czech malting barley harvested from 2008 to 2011, DON occurred the most frequently, with a peak during 2009 [8]. DON, ZON and nivalenol (NIV) predominated in the UK malting barley samples collected between 2007 and 2009 [9]. Barley samples harvested from different regions of Navarra, Spain, during 2008–2009 were contaminated with two or more *Fusarium* toxins [10]. The most frequent combination of DON plus its precursors 15-ADON and 3-ADON was found in 59% of the samples. In Italy, major monitoring efforts have been focused on durum wheat, in which the fungal community structure has been found to be composed of *Microdochium nivale*, *Fusarium poae*, *F. verticillioides*, *F. langsethiae* and *F. graminearum* [11,12]; the most frequently occurring mycotoxin was DON. In Italian environments, preliminary monitoring activities on small grain cereals showed that barley was less contaminated by type-B trichothecenes in comparison with durum wheat. However, among small grain cereals, oats and barley were found to be particularly prone to contamination by type-A trichothecenes. Trichothecenes are sesquiterpenoid molecules characterized by the presence of a tetracyclic 12,13-epoxy ring responsible for the toxicological properties [13]. *Fusaria* produce type A and B trichothecenes: group A includes T-2, HT-2 toxins and diacetoxyscirpenol, whereas group B includes deoxynivalenol and nivalenol. From the surveys cited above, type-B trichothecenes seem to be prevalent in several European environments. However, it is difficult to make generalizations because mycotoxin classes and levels can vary considerably depending on several factors, such as climatic conditions, agronomic factors and host genotype that can lead to changes in the *Fusarium* profile [14]. Type-A trichothecenes have higher toxicity than those of the type-B group and, in particular, T-2 and HT-2 toxins are the most toxic and therefore deserve special attention.

Focusing attention on malting barley, Euromalt—representing the EU malting industry—has reported that the T-2 toxin incidence in malting barley increased during the 2004–2007 period of monitoring [15]. Even if most of the reports for T-2 and HT-2 toxins come from the Scandinavian countries, France and UK [16], it cannot be excluded that these toxins can occur even in other European environments. Indicative levels for the sum of T-2 and HT-2 toxins in cereals and cereal products have been recently issued by the European Commission in the Recommendation 2013/165/EC [17]. An indicative level of 200 µg/kg was established for barley, including malting barley. The aim of this work was to explore the presence of T-2 and HT-2 toxins in barley grown in Italy, as representative of a Southern European environment. More in detail, the T-2 and HT-2 contamination level has been evaluated in Italian barleys during the growing seasons of 2011–2014. The fungal species responsible for the production of these toxins were identified in samples contaminated by T-2 and HT-2 toxins.

## 2. Results and Discussion

This survey aimed to monitor the occurrence of T-2 and HT-2 toxins in malting barley samples collected in the 2011–2014 growing seasons from fields located in regions of Italy that are climatically different and representative of the whole Italian barley cropping area. From our survey, with a total of 691 samples, T-2 and HT-2 occurrence has been found in barley samples coming from all the areas and from all the growing seasons, as summarized in Table 1. The percentage of positive samples (i.e., with a T-2 + HT-2 content > LOQ, Limit Of Quantification) found in the four growing seasons ranges from 22% to 53%. The highest mean (264 µg/kg) and median (212 µg/kg) were observed in samples from 2013, which showed the highest levels of contaminations, with values up to 787 µg/kg. In 2011 and 2012, the positive samples exceeding the EU indicative level of 200 ppb were respectively 2.7% and 2% of the total number of samples. In 2013 and 2014, the percentages of such samples increased respectively to 11.4% and 19.6%. More in detail, 49.6% of the positive samples from 2013 exceeded the 200 µg/kg level, whereas among the 2014 positive samples 37% exceeded this level.

**Figure 1:**
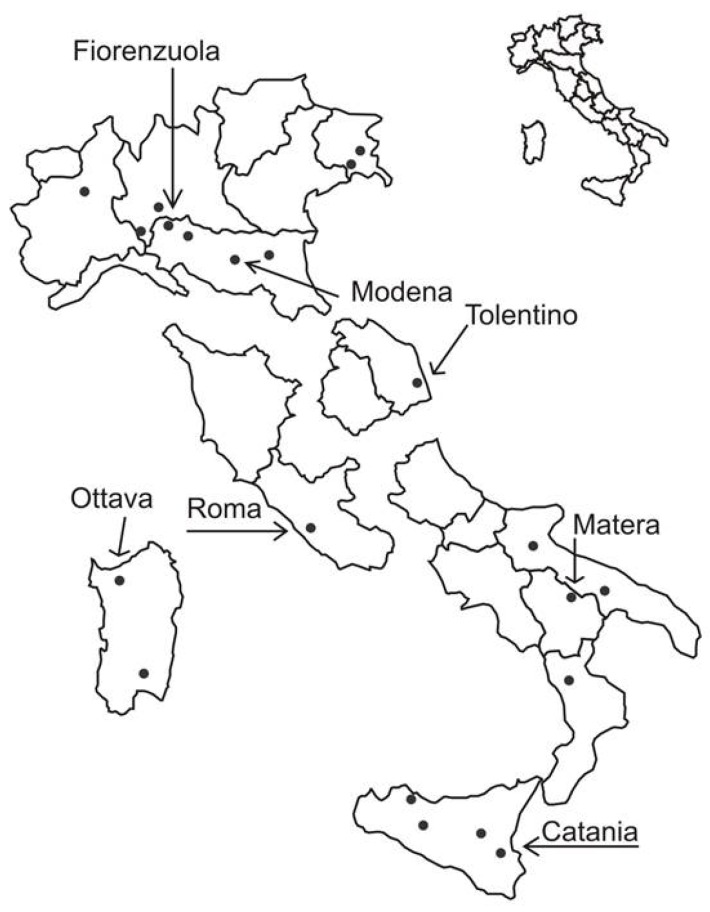
The Italian territory split in the four climatic environments (North, Mideast, Midwest, South + Sardinia and Sicily). Barley samples were collected from the Italian localities indicated by dots, belonging to different climatic areas. The localities in which highly positive samples were found are named.

Significant differences among environments for the presence of barley contaminations were found with the current survey. In particular, the following areas were identified as “hot spots” of T-2 + HT-2 contamination: Fiorenzuola d’Arda and Modena in the North of Italy, Tolentino in the Mideast, Roma in the Midwest, Matera in the South, Catania in Sicily and Ottava in Sardinia (Figure 1).

In Fiorenzuola d’Arda, high incidence and contamination levels were found in 2013 and 2014 (Table 2). In this environment, during the three growing seasons of 2011, 2013 and 2014, the positive samples ranged from 92% to 100%. Only in 2012 was a lower percentage (59%) of positive samples recorded. The Fiorenzuola samples were evaluated also for DON content and an inverse correlation was found between the presence of T-2 and HT-2 and of DON. In fact, only two of the T-2 and HT-2 positive samples also tested positive for DON. The presence of *F. graminearum* and *F. culmorum* was molecularly detected in these two samples. The unlikely co-occurrence of these toxins has been previously observed in Spanish barley as well [10]. Our results are in agreement even with Edwards et al. [18] and Hietaniemi et al. [19]: in fact, these authors observed a mutual exclusion of T-2 + HT-2 and DON. In oats, barley and wheat when T-2 + HT-2 concentration is high, DON concentration is low and vice versa.

In the Modena environment, barley was sown only in 2014 and, in this year, 100% of the samples resulted in contamination, with a mean value of 443 µg/kg, a median value of 481 µg/kg and a range of T-2 + HT-2 content from 137 to 724 µg/kg.

In Tolentino, located in the Mideast, both sowing dates (autumn and spring) were adopted. In this environment, contaminations were recorded during the growing seasons of 2013 and 2014 (Table 3). Interestingly, very different levels of contamination were found between spring and autumn sowing-derived samples. The highest T-2 + HT-2 levels were detected in “spring” samples in both years (Table 3). Analysis of variance (ANOVA) results indicated a statistically highly significant (*p* < 0.001) interaction between year, sowing date and variety. This result points to the putative cause of interaction between plant phenology and climatic conditions favourable for dispersal, growth of the fungi and their mycotoxins production.

In Roma (Midwest) only two spring sown varieties (Overture and Scarlett) resulted in contamination in 2014. Surprisingly, in 2014, positive samples were found even in southern localities, traditionally considered environments free from mycotoxin contamination. All samples collected in Matera were found to be positive, with a mean value of 165 µg/kg, a median value of 157 µg/kg and a range of T-2 + HT-2 content from 29 to 324 µg/kg. In Catania (Sicily), 21% of the samples collected in 2014 were positive (mean value of 26 µg/kg, median value of 5 µg/kg and a range of 28–258 µg/kg in positive samples). In Sardinia (Ottava), during 2014, 86% of the samples were positive (mean 57 µg/kg, median 30 µg/kg and a range of 25–238 µg/kg in positive samples).

**Figure 2:**
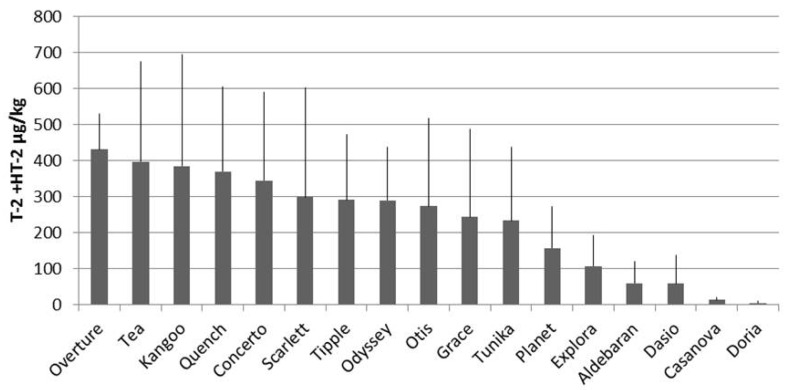
Mean levels (plus standard deviations) of T-2 + HT-2 in grains of malting- and feed-quality barley varieties harvested in Fiorenzuola d’Arda and Tolentino in 2012, 2013 and 2014.

Focusing attention on the cultivar performance in Fiorenzuola d’Arda and Tolentino spring field trials, a wide variability has been observed for T-2 + HT-2 levels. Figure 2 shows the mycotoxins mean content in the grains of the indicated varieties grown in these two environments in the growing seasons of 2012, 2013 and 2014. A clear gradient of contamination can be observed among different varieties sown in spring in each growing season. Among the less contaminated there are the majority of “control” feed varieties, such as Doria, Dasio, Aldebaran and Explora. All the malting varieties were strongly contaminated, with the exception of Casanova. However, very different levels of mycotoxin contamination were observed in all these cultivars after autumn or spring sowing in Tolentino, again suggesting a major role of phenology in the success of fungal infection. To gain more information on this point, a set of five varieties autumn sown in Fiorenzuola d’Arda during 2011, 2012 and 2013 were analyzed for T-2 + HT-2 content.

**Figure 3:**
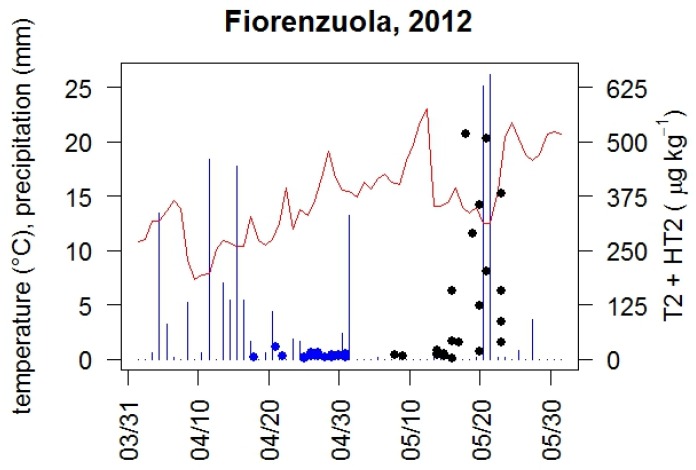
T-2 + HT-2 levels (μg/kg) versus heading date, daily precipitation (blue bars), and average temperature (red line) in 2012. Results for both autumn-sown (blue circles) and spring-sown (black circles) plants are shown.

The example of the year 2012 (Figure 3) shows that early flowering winter-sown plants and some early flowering spring-sown varieties did not carry detectable mycotoxin loads. Grains of later heading (second half of May) spring-sown plants carried detectable mycotoxin loads. The time window around heading of those varieties was characterized by episodes of short but intense rainfall and increasing temperatures. The environmental temperatures and the relative humidity have been suggested to have a role in T-2 and HT-2 barley contamination in several cultivation areas. Opoku et al. [20] associated the contamination with warm periods and high relative humidity in the UK. Linkmeyer et al. [21] found a positive correlation between T-2 and HT-2 producer *Fusaria* occurrence and temperature increase in the Bavarian environment. The FinMyco survey [19] evaluated the percentages of *Fusarium* infection in wheat, barley and oats in Finland in 2005–2006 and found higher levels of infection in all spring cereals in 2005 in comparison with 2006. Interestingly, 2006 was a much drier year than 2005, that was characterized by heavy rainfalls immediately after flowering days. Rossi et al. [22] found an association between rainfall and peaks of *Fusarium* macroconidia sampled from the air in wheat fields naturally affected by Head Blight. In particular, the number of conidia progressively increase during rainfall and can be found still at high densities for some hours after the rain has ceased. Orlando et al. [23] hypothesized that the infection of barley by T-2 producing *Fusaria* requires the conidial dispersal of the fungus to coincide with host flowering. In particular, the end of heading was found decisive in determining spike infection. The infection of cereal heads before flowering was identified by Opoku et al. [20] as one of the major difference between the infection process of T-2 producing *Fusaria* and the other FHB (Fusarium Head Blight) pathogens. In Italian environments, barley plants often flower at the time of ear emergence and the flowering lasts only for a few days. Late flowering plants could therefore be colonized by the asexual conidia produced and dispersed later in the season.

As the final result of this complex interaction, in our survey, higher contamination of toxins was found in spring-sown samples and these findings are in agreement with data observed in small grain cereal samples from other European countries like France and Lithuania [23,24]. Even in Finland, spring cereals have been noted to be more susceptible to fungal infection and mycotoxins formation than winter cereals [19]. Orlando et al. [23] identified sowing date as the most important agronomic factor that influences the levels of T-2 and HT-2 toxins in French barley. Moreover, several other factors can play a role in the *Fusarium*-barley interaction, including plant genetic makeup. FHB resistance has been described as a very complex quantitative trait that can be broken down into at least five different components: resistance to initial infection; to pathogen spread; to toxin accumulation; to kernel infection, and tolerance. Active factors of resistance include physiological processes that are activated by the plant and influence host colonization by the fungus. Passive factors can be morpho-physiological traits, such as plant height, spike and flower architecture and flowering date [5]. Genetic resistance/susceptibility can therefore be an additional factor contributing to determine the differences among samples, as demonstrated by the significant interaction between year, sowing date and variety observed in Tolentino samples. One aim of our survey was to identify the occurrence and correlation of *Fusarium* species present in Italian malting barley samples and T-2 + HT-2 toxins. The presence of A and B trichotecenes *Fusaria* producers were evaluated, using species-specific molecular assays, in all the samples (57) with T-2 + HT-2 at levels >200 µg/kg and in an additional 20 samples with contamination levels ranging from 0 to 200 µg/kg. The presence of DON producers *F. graminearum* and *F. culmorum* and of the T-2 + HT-2 producer *Fusarium sporotrichioides* was not detected. On the contrary, *F. langsethiae* was present in 100% of the T-2, HT-2 positive samples, coming from all the tested environments. A significant correlation (*r* = 0.719***) was found between the amount of T-2 + HT-2 toxins and the quantity of *F. langsethiae* DNA. *F. poae* was detected in 60% of the analyzed samples, however, a nonsignificant correlation (*r* = 0.377) was found between the amount of T-2 + HT-2 toxins and the amount of *F. poae* DNA. These findings are in accordance with the results of Yli-Mattila et al. [25,26,27], Fredlung et al. [28], Wilson et al. [29] and Edwards et al. [30]. In oats, all these authors found a positive correlation between *F. langsethiae* DNA amounts and T-2 + HT-2 levels. On the contrary, *F. poae* DNA concentration was found uncorrelated with HT-2 + T-2 concentration. Edwards et al. [30], on the basis of multiple regression analysis with *F. langsethiae* and *F. poae* DNAs as explanatory variates, indicated that higher concentrations of *F. poae* DNA were correlated with slightly lower concentrations of HT-2 + T-2. Therefore, they concluded that “*F. langsethiae* is the primary, if not sole, fungus responsible for high HT-2 and T-2 in UK oats”. It is likely that all these authors could not find the same correlation in barley due to the lower levels of T-2 + HT-2 toxins detected in their environments. To better clarify the role of *F. poae* in mycotoxin accumulation in our barley samples, an additional experiment was done. Starting from T-2 + HT-2 contaminated grains of three different varieties (Concerto, Scarlett and Overture) coming from two different environments (Fiorenzuola and Modena), *F. poae* strains were isolated. These *F. poae* isolates were characterized for T-2 + HT-2 in vitro production in comparison with a *F. langsethiae* reference strain. It was found that *F. langsethiae* has a mean T-2 + HT-2 in vitro production of 396 ppb, whereas the T-2 + HT-2 productions of *F. poae* strains are under the LOQ of the ELISA test. The failure of *F.* *poae* isolates to produce T-2 + HT-2 toxins is in accordance with the results of Jestoi et al. [31,32] and Kokkonen et al. [33]. In vivo and in vitro studies have shown that *F. poae* very rarely produces type A trichothecenes and instead produces quite high amounts of beauvericin, enniatins, fusarenon-X, diacetoxyscirpenol and nivalenol in cereals [34]. From the collected evidence, we can conclude that *F. langsethiae* was the fungus mainly responsible for T-2 + HT-2 accumulation in Italian malting barleys evaluated in our survey. The presence of mycotoxigenic *F. langsethiae* in Italy has previously been reported only for durum wheat samples harvested in 2006 and, more recently, in 2014 [12,35]. Therefore, this is, to the best of our knowledge, the first report of T-2 and HT-2 occurrence due to *F. langsethiae* in malting barley cultivated in a Mediterranean environment. Additionally, Hofgaard et al. [36] identified *F. langsethiae* as the main T-2 and HT-2 producer in oats and wheat in Norway over a six-year period. This fungus, described by Torp and Langseth [37] as “A *Fusarium* species with a micro morphology similar to *F. poae* and a metabolite profile resembling that of *F. sporotrichioides”* was later identified as a new species, named *Fusarium langsethiae* [38]. A draft genome sequence of this fungus has been recently released [39] and it was demonstrated that the identified putative *Tri5* cluster of *F. langsethiae* is highly synthenic to the cluster reported in *F. sporotrichioides*. The chemical profiling showed that *F. langsethiae* produces a high number of secondary metabolites, dominated by type A trichothecenes.

In our survey, no visual symptoms of fungal infection were detected in any of the analyzed kernels and grain yield was not affected. No significant correlation was found between toxin levels (µg/kg) and yield (tons/ha) across the trials grown in Fiorenzuola d’Arda (2013 and 2014), Tolentino (spring sowing, 2013 and 2014) and Modena (2014).

Our observations are in agreement with previous findings in other small grain cereals, like oats and wheat [40,41,42]. In glasshouse experiments on oats and wheat, *F. langsethiae* infection did not produce FHB symptoms, behaving as a symptomless, weak pathogen [43]. Nazari et al. [42], in durum wheat infected with *F. langsethiae*, observed “no relationship between DNA or mycotoxin content and incidence of shrivelled or discoloured kernels or grain yield components”. Moreover, in the open field, *Fusarium langsethiae* did not interfere with oat yield [44].

The presence of *F. langsethiae* and T-2 and HT-2 toxin contamination in symptomless kernels of barley is important for food and feed safety because the lack of visual symptoms does not mean that the grains are free from these mycotoxins. This asyntomaticity can be considered an additional factor of risk for accidental exposure to T-2 and HT-2, which, among trichothecenes, are considered the most acutely toxic members. Several in vitro and in vivo studies have identified the key effects of T-2 and HT-2 in eukaryotic cells and in animal model systems, as reviewed by Rocha et al. [45], Escriva et al. [46], De Ruick et al. [47], Gallo et al. [48] and reported in the “Scientific Opinion on the risk for animal and public health related to the presence of T-2 and HT-2 toxin in food and feed” [49]. At the cellular level, the major effect of T-2 is the inhibition of protein synthesis, as first reported by Ueno [50] and, consistently with this primary effect, the nucleic acid synthesis and the mitosis are affected. T-2 toxin induces cell apoptosis, mitochondrial functions alterations and cellular membrane damage due to lipid peroxidation. The major effect at the animal organism level is immunosuppression, however, several other problems are caused by T-2 in vivo, among others growth retardation, reproductive disorders, gastric lesions, myelotoxicity.

In our monitoring, we have observed an increasing number of contaminated areas in Italy during the 2011–2014 period; this fact likely indicates an enrichment of *F. langsethiae* spores in these Mediterranean environments. From the breeder’s point of view, our observations strongly suggest that the evaluation of susceptibility level of a barley line or variety must be done taking into account several factors, including the year, the location and the sowing date. Moreover, because spring sowing seems to be an additional risk factor, a major target for malting barley breeding—at least for Mediterranean environments—can be the development of genotypes with winter or alternative habitus of growth for autumn sowing.

Our observations indicate the need for more detailed studies aimed to a better understanding of the *F. langsethiae* population’s ecology and of the environmental conditions associated with the successful growth of the fungus and mycotoxin production. To date, in fact, only a few studies have focused on the characterization of *F. langsethiae* biology, examples being the works of Kokkonen et al. [51] and Nazari et al. [42], in which the temperature and humidity requirements of this fungus have been determined both in vitro and in vivo. Strub et al. [52] found that the optimal temperature and water activity for *F. langsethiae* toxinogenesis are 28 °C and 0.997. Hofgaard et al. [53] found that, contrarily to what observed for other *Fusaria*, low levels of *F. langsethiae* were detected in the cereal residues at the soil surface as a result of reduced tillage practices, therefore the main inoculum source for *F. langsethiae* remains unclear.

## 3. Conclusions

This is the first report of the occurrence of *F. langsethiae*—and of its toxic metabolites T-2 and HT-2 toxins—in malting barley grown in Italian environments. The T-2 + HT-2 mean values (calculated on positive samples) and the maximum values observed in some Italian environments are high, in comparison with contamination levels found in barley from different European regions, as reported in Table 4. Data presented herein suggest an increasing mycotoxin risk for the Italian barley-malt production chain. The lack of visual symptoms observed on contaminated barley kernels is an additional food and feed safety issue. A deeper understanding of *F. langsethiae* biology and epidemiology is needed in order to develop measures directed towards minimizing the barley infection and subsequent contamination with mycotoxins.

## 4. Material and Methods

### 4.1. Collection of Barley Samples

Barley samples used in this work were obtained from the “Italian Network of Variety Performance Trials”, which is annually funded by the Italian Ministry of Agriculture to provide update information to operators about agronomic characteristics, yield and commercial quality of the new and most widely cultivated varieties, suggesting the choice of varieties for different cultivation areas. Agronomic protocols adopted in the network are comparable with those routinely used by the farmers in conventional practice, therefore the data obtained are reliable even for commercial productions. Trials were laid out in a randomized complete block design with three replicates and were organized in different areas representing the whole Italian cropping territory (Figure 1). In this territory, four climatic regions can be distinguished: North (cold winter: −1/+2 °C average temperature during coldest month, humid area); Mideast (mild winter: +4/+6 °C, humid); Midwest (mild winter: +6/+8 °C, humid) and South + Sardinia and Sicily (mild winter: +6/+8 °C, occasionally dry). In the Northern localities fields were sown, as is customary in these environments, in spring, whereas the Southern ones were sown in autumn. In the Central regions, fields were sown in both seasons. Four harvests (2011, 2012, 2013 and 2014) were considered for the survey. The following malting varieties were included each year in the trials: Concerto, Grace, Orchidea, Otis, Pariglia, Scarlett, Tipple, Tunika. Braemar was sown only in 2011, whereas Casanova and Merveil, in 2011 and 2012; Kangoo in 2011, 2012 and 2013; Odyssey in 2013 and 2014, Overture, Quench and Planet in 2014. The following feed quality varieties were also included, as reference for yield evaluation: Aldebaran, Dasio, Doria, Explora, Tea.

A total of 691 samples of barley was collected from the harvests 2011, 2012, 2013 and 2014. At maturity (13% humidity of the grains), all the plants of each accession (10 m^2^ plots, three repetitions) were harvested and threshed without further treatments. Each plot produced 3 to 8 kg of grains, depending on year, locality and variety. We have considered as “lot” all the grains produced by the plots in three repetitions. The total grain production of the three repetitions ranged from 9 to 24 kg and therefore we have considered it as “very small lot” (EC sampling regulation 401/2006). Through a static procedure (manual coning and quartering), three incremental samples of 350 g were obtained from the three plot repetitions. The three incremental samples were mixed in an aggregate sample of 1 kg weight. This grain quantity is representative of 500–600 barley spikes. A similar number of ears has been suggested as useful to estimate the true field concentration of Aflatoxin B_1_ in corn [58]. This aggregate sample was dry milled into a fine powder having a particle size of 1.0 mm using an analytical mill (IKA Universal mill M20, IKA-Werke GmbH, Staufen, Germany) and stored at 4 °C until analysis. A laboratory sample of 50 g was obtained from each aggregate sample after manual coning and quartering and used both for mycotoxin analysis and for DNA extractions.

### 4.2. Fungal Strains

Table 5 reports some information about the *Fusarium sporotrichioides*, *F. langsethiae*, *F. poae*, *F. graminearum* and *F. culmorum* monosporic reference strains used in the work. These fungal strains were grown in PDA (Potato Dextrose Agar) medium and the DNAs extracted from the mycelia (procedure reported below) were used to build calibration curves in real-time PCR (Polymerase Chain Reaction) assays.

Additional *F. poae* isolates were prepared starting from a set of malting barley samples (cv. Concerto, Scarlett and Overture) coming from the field trials of Modena and Fiorenzuola d’Arda (2014) and found contaminated with T-2 and HT-2 (this paper). Starting from about 100 seeds of each barley sample, *F. poae* isolates were obtained according to Infantino et al. [12]. The isolates were first identified by microscope observation and then the species identity has been confirmed with qPCR assays, as described below (“Determination and quantification of *Fusarium* species” section).

### 4.3. Mycotoxin Analysis

Barley grains were ground into a fine powder having a particle size of 1.0 mm using an analytical mill as previously described. Competitive direct immunoassays ELISA have been used for mycotoxin detection. The choice of this analytical method has been done for economic reasons. The amount of T-2 and HT-2 toxins (as sum of toxins) was determined by using the kit Veratox^®^ T-2/HT-2 (Neogen Corporation, Lansing, MI, USA) according to the manufacturer’s instructions. The intended use of this kit, according to the manufacturer, is in commodities such as wheat, corn, barley, oats and rye. The lower limit for toxins detection (LOD, Limit of Detection) for this assay is 25 µg/kg, whereas its range of quantification is 25–250 µg/kg. The sample extracts exceeding the 250 µg/kg have been further diluted, reanalyzed and the dilution factor has been included when the final results have been calculated. A validation trial has been organized to evaluate the Veratox^®^ T-2/HT-2 performance in comparison with UPLC-MS/MS (Ultra-performance liquid chromatography tandem mass-spectrometry) [63]. CREA-GPG laboratory partecipated to the trial. The amount of DON was determined by using the Ridascreen^®^DON competitive enzyme immunoassay kit (R-Biopharm AG, Darmstadt, Germany) according to the manufacturer’s instructions. The declared lower limit of detection for this assay is 3.7 µg/kg and the range of quantification is 3.7–100 µg/kg. All samples were analyzed in triplicate.

*F. poae* isolated from Modena and Fiorenzuola grown barley samples (see section “Fungal strains”) and reference isolate of *F. langsethiae* were cultured in GYEP (Glucose Yeast Extract Peptone), (5% glucose, 0.1% yeast extract, 0.1% peptone) liquid medium as described by Busman et al. [64]. The amount of T-2 and HT-2 toxins was determined on the cultures using the kit Veratox^®^ T-2/HT-2 (Neogen Corporation, Lansing, MI, USA).

### 4.4. Determination and Quantification of Fusarium Species

#### 4.4.1. DNA Extractions 

Plant genomic DNA was extracted from 300 mg of ground barley grains. This subsample was obtained from the 50 g laboratory sample described in the “Collection of barley samples” section by picking three times 100 mg and weighing with a Mettler-Toledo AT261 (Mettler-Toledo S.p.A., Novate Milanese, Italy) balance. The size of the subsample is congruent with Sarlin et al. [65], who evaluated the repeatability of the DNA extraction method for the real-time PCR-based quantification of toxigenic *Fusarium* species in barley and malt. The 300 mg of ground barley grains was mixed with 860 μL of extraction buffer (10 mM Tris-HCl, pH 8.0, 150 mM NaCl, 2 mM EDTA, 1% SDS) and 100 µL of guanidinium chloride. After incubation at 60 °C for 3 h, the samples were centrifuged for 10 min at 12,000× *g*. A total of 5 μL of RNase (500 μg/μL) was added to 500 μL of the supernatant and incubated at 37 °C for 10 min to digest the contaminating RNA. The extracted DNA was purified using the Wizard^®^Genomic DNA Purification Kit (Promega Corporation, Madison, WI, USA) according to the protocol suggested by the manufacturer for plant tissues and eluted with 50 μL buffer (10 mM Tris-HCl, pH 9.0). *Fusarium* mycelium was lyophilized as described by Terzi et al. [61]. Fungal DNAs were extracted from the lyophilized mycelia as described by Al-Samarrai and Schmid [66].

Plant and fungal DNA concentrations and quality were determined after photometric-readings at 260 and 280 nm (NanoDrop 1000, Thermo Fisher Scientific, Wilmington, DE, USA).

#### 4.4.2. Real-Time PCR

Table 6 reports the information on the PCR primers used. Real-time reactions were prepared with 10 μL of KAPA SYBR FAST qPCR Master Mix (KAPA BIOSYSTEMS, Boston, MA, USA), 900 nM forward and reverse primers, 50 ng of DNA template and water to 20 μL. All PCR samples and controls were prepared in duplicate. PCR was performed on a 7300 Real Time PCR System (Applied Biosystems, Thermo Fisher Scientific, Waltham, MA, USA) using the following cycling protocol: 95 °C for 3 min; 40 cycles of 95 °C for 3 s and 60 °C for 30 s. For the quantification of *Fusarium* DNA a standard curve was generated by plotting the Ct (Cycle Threshold) values versus the log10 amount of pure DNA of the different *Fusaria* (10-fold dilution series). The quantification of fungal DNA in barley samples was derived from the standard curve run in parallel reactions and the results were expressed as ng of fungal DNA starting from 50 ng of total DNA (of plant and fungal origin) extracted from a sample. A melting curve analysis step was always included in each run to control for false-positive results caused by primer-dimer hybridization and non-specific amplifications.

### 4.5. Meteorological Data

Meteorological data were available from two meteorological stations located within the experimental farm and were used to produce a homogenized complete data series. Average daily temperature and daily rainfall were used for characterizing the climatic conditions associated with mycotoxin loads.

### 4.6. Statistical Analyses

Analysis of variance—ANOVA—was performed with the SYSTAT Version 12.0 package (Cranes Software International Ltd., Bangalore, India, 2005). Those *p*-values < 0.05 were considered to be statistically significant.

Pearson’s correlation coefficients were calculated with the SYSTAT 12.0 package to determine the degree of association between variables. The original DNA and toxin concentrations were transformed to logarithmic values in order to obtain a more normal distribution for the values of toxin and DNA concentrations [19].

## Figures and Tables

**Table 1:** Incidence and levels of contamination with T-2 and HT-2 toxins (as sum) in barley grain samples from Italy in the growing seasons 2011, 2012, 2013 and 2014.

Growing Season	Number of Analyzed Samples	Positive Samples ^a^				
		%	Mean (µg/kg)	Median (µg/kg)	Range (µg/kg)	% Positives > 200 µg/kg ^b^
2011	72	28	69	54	30–467	2.7
2012	303	22	72	34	26–518	2
2013	158	23	264	212	26–787	11.4
2014	158	53	106	27	26–724	19.6

^a^ All samples with value > Limit of Quantification (LOQ) were considered to be positive (LOQ = 25 μg/kg for T-2 + HT-2); ^b^ EU indicative levels for barley (European Commission 2013).

**Table 2:** Incidence % and levels of contamination of T-2 and HT-2 toxins (as sum, μg/kg) in barley grain samples from Fiorenzuola d’Arda (PC), Italy, in the growing seasons of 2011, 2012, 2013 and 2014.

Growing Season	Number of Analyzed Samples	Positive Samples ^a^			
		%	Mean	Median	Range
2011	28	100	95	66	37–467
2012	48	59	70	40	39–518
2013	18	94	358	285	90–787
2014	13	92	271	276	76–466

^a^ All samples with value > LOQ were considered to be positive (LOQ = 25 μg/kg for T-2 + HT-2).

**Table 3:** Incidence % and levels of contamination of T-2 and HT-2 toxins (as sum, μg/kg) in barley grain samples from Tolentino (MC), Italy, in the growing seasons of 2012, 2013 and 2014.

Growing Season	Number of Analyzed Samples	Positive Samples ^a^			
		%	Mean	Median	Range
2012	13	0	-	-	-
2013, autumn sowing	11	27	25	22	29–64
2013, spring sowing	9	73	312	296	57–877
2014, autumn sowing	15	71	30	29	26–36
2014, spring sowing	13	70	109	76	43–391

^a^ All samples with value > LOQ were considered to be positive (LOQ = 25 μg/kg for T-2 + HT-2).

**Table 4:** T-2 and HT-2 contamination levels detected in barley samples from different regions in Europe.

Toxin	Mean Value (μg/kg)	Max Value (μg/kg)	Country	Period	Reference
T-2 + HT-2	36	277	Finland	2005–2006	[19]
T-2 + HT-2	37	626	Finland	2005–2014	[19]
T-2 + HT-2	≤20	-	Norway	2002–2004	[54]
T-2	30	-	Czech Republic	2005–2008	[55]
HT-2	110	716	Czech Republic	2005–2008	[55]
T-2 + HT-2	8.9	145	Czech Republic	2008–2011	[8]
T-2 + HT-2	29.2 (in positive samples)	532.5	Spain	2007	[10]
T-2 +HT-2	≤20	138	UK	2002–2005	[18]
T-2	19.7	319	Lithuania	2003–2005	[56]
T-2 + HT-2	45.72	755.7	France (spring barley)	2007–2010	[57]
T-2 + HT-2	10.4	340.1	France (winter barley)	2007–2010	[57]
T-2 + HT-2	127 (in positive samples)	787	Italy	2011–2014	this work

**Table 5:** Details on the fungal strains used in the work: In the table are indicated some works in which the strains have been already used.

Species	Strain	Source of Isolation	Obtained from	Repository	References
*F. sporotrichioides*	ITEM 194	*Zea mays* kernel (Italy)	Institute of Sciences of Food Production, CNR, Bari, Italy	Institute of Sciences of Food Production, CNR, Bari, Italy	[59]
*F. langsethiae*	11020	Durum wheat kernels (Italy)	Università del Sacro Cuore, Di.Pro.Ve.S., Piacenza, Italy	Università del Sacro Cuore, Di.Pro.Ve.S, Piacenza, Italy	[42]
*F. graminearum*	ITEM 6477	*Triticum* sp. (Italy)	Institute of Sciences of Food Production, CNR, Bari, Italy	Institute of Sciences of Food Production, CNR, Bari, Italy	[60]
*F. culmorum*	MPVP 70	Bread wheat plants (Italy)	Università del Sacro Cuore, Di.Pro.Ve.S, Piacenza, Italy	Università del Sacro Cuore, Di.Pro.Ve.S, Piacenza, Italy	[61]
*F. poae*	ITEM 10402	Wheat kernels (Italy)	Università del Sacro Cuore, Di.Pro.Ve.S, Piacenza, Italy	Institute of Sciences of Food Production, CNR, Bari, Italy	[62]

**Table 6:** Details on the PCR (Polymerase Chain Reaction) primers used in the work.

Primer Names and Sequences	Use	Reference
TC130707F (TCGGCTACAGCATTGAAGACG)	Barley DNA quantification	[67]
TC130707R (CCAAAAACGATATCAGGATGGC)		
F.langA29F (CAAGTCGACCACTGTGAGTACCTCT)	*F. langsethiae* DNA quantification	[68]
F.langA95R (TGTCAAAGCATGTCAGTAAAGATGAC)		
F.spoA18F (CGAAGTCGACCACTGTGAGTACA)	*F. sporotrichioides* DNA quantification	[68]
F.spoA18R (CTGTCAAAGCATGTCACTAAAAATGAT)		
F.poaeA51F (ACCGAATCTCAACTCCGCTTT)	*F. poae* DNA quantification	[68]
F.poaeA98R (GTCTGTCAAGCATGTTAGCACAAGT)		
22F (AATATGGAAAACGGAGTTCATCTACA)	*F. graminearum* and *F. culmorum* DNA quantification	[68]
122 R (ATTGCCGGTGCCTGAAAGT)		
